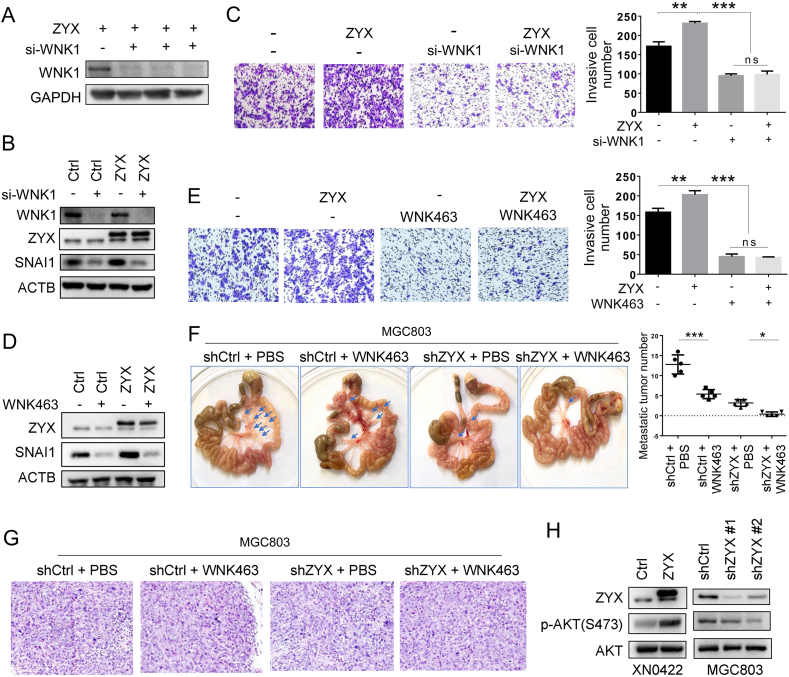# ZYX promotes invasion and metastasis of gastric cancer cells via WNK1/SNAI1axis

**DOI:** 10.1016/j.gendis.2023.03.018

**Published:** 2023-04-13

**Authors:** Yi Jiang, Dongfang Xiang, Xianmei Wen, Mingmin He, Yan Qin, Xiaoxue Yao, Zexuan Yan, Xiuli Geng, Yong Ren, Xiongwei Cai, Youhong Cui, Yan Wang

**Affiliations:** a Institute of Pathology and Southwest Cancer Center, Southwest Hospital, Army Medical University (Third Military Medical University), Chongqing 400038, China; b Department of Pathology, General Hospital of Central Theater Command of PLA, Wuhan, Hubei 430070, China; c Department of Obstetrics and Gynecology, Southwest Hospital, Army Medical University (Third Military Medical University), Chongqing 400038, China; d Department of Pharmacy, The Hospital of 83rd Group of PLA, Xinxiang, Henan 453000, China; e Department of Gynecology, Chongqing Health Center for Women and Children, Women and Children's Hospital of Chongqing Medical University, Chongqing 400013, China

Gastric cancer (GC) is the third most common cause of cancer death globally and a large portion of patients are diagnosed at advanced stages with cancer invasion and metastasis^1^^,^^2^. However, the mechanisms underlying the invasion and metastasis of GC remain to be delineated. ZYX plays critical roles in cell mobility via cytoskeleton regulation in various cell types.^3^ In this study, we further reported that ZYX promoted migration, invasion, and metastasis of GC cells. Mechanistically, ZYX promoted WNK1 activation and SNAI1 up-regulation, inducing epithelial-mesenchymal transition (EMT) to enhance the mobility of GC cells. Inhibition of WNK1 impaired the mobility of GC cells. Therefore, ZYX/WNK1 could be potential therapeutic targets for GC treatment.

Immunohistochemistry (IHC) in a GC cohort showed that the protein level of ZYX in GC tissues was significantly higher than that in paracancerous tissues (Fig. 1A; Fig. S1A). The statistical analysis on the TCGA_STAD database and GSE2685, a GC dataset from GEO, consistently showed that the mRNA level of ZYX gene in GC cells was higher than that in non-tumor counterparts (Fig. S1B, C). In 6 pairs of fresh GC tissues and paracancerous tissues, we also observed that the protein and mRNA levels of ZYX were increased in GC tissues compared with paracancerous tissues (Fig. S1D, E). Then, we analyzed the correlation between ZYX level and clinicopathological parameters of GC patients. The results showed that the expression of ZYX was positively correlated with tumor size, recurrence, and T and N stages (Table S1). One-way ANOVA and multivariate ANOVA analyses showed that ZYX was an independent prognostic factor (*P* < 0.0001) for GC patients (Table S2). The overall survival (OS) time and disease-free survival (DFS) time of GC patients with ZYXHigh were significantly shorter than those of ZYXLow patients (Fig. 1B). Analysis of the GC database from Kaplan–Meier Plotter further supported the prognostic significance of ZYX (Fig. S1F).

**Figure 1:**
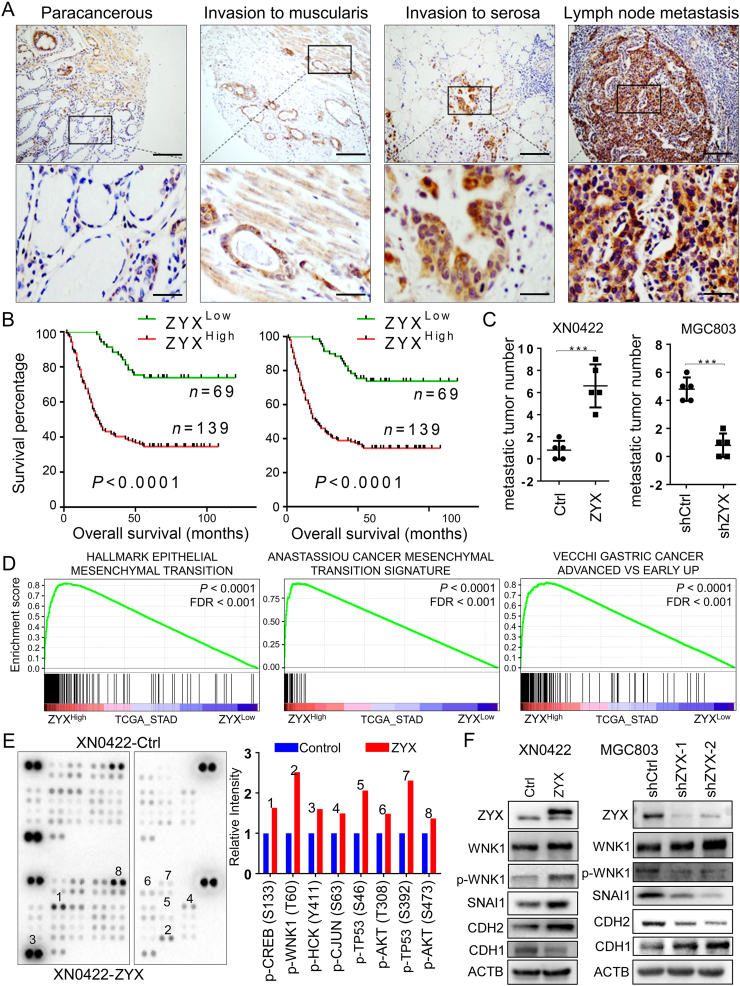
The expression and regulation of ZYX in GC tissues. **(A)** IHC images of ZYX with different invasion depths and lymph node metastasis in GC. Scale Bar = 200 μm (upper) and 50 μm (lower). **(B)** Kaplan–Meier survival analysis of GC patients with high ZYX versus those with low ZYX. **(C)** Statistic graph of intraperitoneal metastatic nodules in control (Ctrl) versus ZYX-overexpressing (ZYX) mice or control-shRNA (shCtrl) versus ZYX knockdown (shZYX) mice. **(D)** Gene sets enriched by high ZYX versus low ZYX through GSEA. **(E)** Phosphokinase array image and the semi-quantitative result of altered phosphorylated kinases. **(F)** Western blotting of indicated proteins in stable cells based on XN0422 and MGC803 cells.

To study the role of ZYX in GC cells, we measured the expression of ZYX in normal gastric mucosal epithelial cells (GES-1), a panel of GC cell lines (BGC823, MGC803, SGC7901), and a primary GC cell line XN0422 by real-time PCR and western blotting. Both mRNA and protein levels of ZYX were increased in GC cell lines compared to normal cells (Fig. S2A). We constructed ZYX-overexpressing cells using XN0422 (XN0422-ZYX) and ZYX-knockdown cells using MGC803 (MGC803-shZYX) (Fig. S2B, C) to further explore the effect of ZYX on the mobility of GC cells. Wound-healing assay showed that the migration ability of XN0422-ZYX cells was enhanced compared with XN0422 control (XN0422-Ctrl) cells (Fig. S2D). However, the migration ability of MGC803-shZYX cells was reduced compared with MGC803 control (MGC803-shCtrl) cells (Fig. S2D). The invasive ability of XN0422-ZYX cells was significantly higher than that of XN0422-Ctrl cells (Fig. S2E), and the invasive ability of MGC803-shZYX cells was significantly decreased compared to MGC803-shCtrl cells (Fig. S2E). Through abdominal metastasis experiment in mice, we found that the number of metastatic tumors in XN0422-ZYX cells was significantly higher than that in XN0422-Ctrl cells (Fig. 1C; Fig. S2F), but the number of metastatic tumors in MGC803-shZYX cells was significantly lower than that in MGC803-shCtrl cells (Fig. 1D; Fig. S2G). HE staining confirmed that the metastatic tumor originated from GC cells (Fig. S2H), confirming that ZYX enhanced the mobility of GC cells both *in vitro* and *in vivo*.

To explore potential signaling pathways involved in the ZYX-regulated mobility of GC cells, we analyzed GC databases from TCGA_STAD, GSE35809, and GSE2685 via Gene Set Enrichment Analysis (GSEA).^4^ We used the median value of ZYX mRNA level as the cutoff to stratify cases into high (ZYXHigh) and low (ZYXLow). In the context of Hallmark signatures, EPITHELIAL MESENCHYMAL TRANSITION (EMT) had the highest enrichment score (ES) among genesets enriched by ZYXHigh (Fig. 1E and Table S3). In the context of KEGG pathways, ECM RECEPTOR INTERACTION, and FOCAL ADHESION were highly enriched by ZYXHigh (Table S4). Using curated genesets as background, we found that ANASTASSIOU CANCER MESENCHYMAL TRANSITION SIGNATURE and JECHLINGER EPITHELIAL TO MESENCHYMAL TRANSITION UP were enriched by ZYXHigh (Fig. 1E and Table S5). Interestingly, a GC-related geneset VECCHI GC ADVANCED VS EARLY UP was also consistently enriched by ZYXHigh (Fig. 1E and Table S5). It was noted that GSEA results from the three databases were largely overlayed, indicating the reliability of our analysis on ZYX expression (Fig. S3).

To identify potential ZYX-related kinases in GC cells, we examined kinases activated by ZYX using a human phospho-kinase antibody microarray. The results showed that the phosphorylation levels of eight kinases in XN0422-ZYX cells were higher than those in XN0422-Ctrl cells, among which the elevated level of p-WNK1 was the most significant (Fig. 1F). To verify the relationship between p-WNK1 and ZYX, we overexpressed ZYX in BGC823 (BGC823-ZYX) cells (Fig. S4A) followed by screening via phospho-kinase antibody microarray. The result showed that p-WNK1 in BGC823-ZYX cells was also increased compared to BGC823 control (BGC823-Ctrl) cells (Fig. S4B). Consistently, western blotting confirmed the increase and decrease of p-WNK1 level by overexpression and knockdown of ZYX, respectively (Fig. 1G). It has been reported that WNK1 affects the EMT of lung cancer cells through SNAI1 and enhances the mobility of lung cancer cells,^5^ which promoted us to examine the axis of ZYX/WNK1/SNAI1 in GC cells. The results showed that ZYX overexpression up-regulated the protein levels of SNAI1 and CDH2 (N-cadherin), but down-regulated the protein levels of CDH1 (E-cadherin) in XN0422 cells (Fig. 1G). ZYX knockdown in MGC803 cells decreased SNAI1 and CDH2 but increased CDH1 (Fig. 1G). Therefore, ZYX might induce the occurrence of EMT in GC cells through WNK1/SNAI1 pathway.

We further evaluated the relationship between ZYX and SNAI1 using TCGA_STAD and GSE35809 databases. mRNA levels of ZYX and SNAI1 were significantly and positively correlated with each other (Fig. S4C). GSEA showed that mobility-related genesets were consistently enriched by SNAI1 high expression (Fig. S4D–F and Table S6–8). In the TCGA_STAD database, the transcriptomic profile of ZYXHigh and ZYXLow resembled those of SNAI1High and SNAI1Low (Fig. S4G and Table S9). David's analysis showed that the co-upregulated genes by ZYX and SNAI1 were enriched in gensets related to cell mobility regulation (Fig. S4G).

Finally, we examined the effect of WNK1 deletion on the ZYX-induced invasion of GC cells. Knockdown of WNK1 (Fig. S5A) remarkably decreased the expression of SNAI1 (Fig. S5B) and suppressed the invasion of XN0422-ZYX cells (Fig. S5C). Treatment with WNK463, an inhibitor of WNK1, led to a decrease in SNAI1 protein level in both XN0422-ZYX and XN0422-Ctrl cells (Fig. S5D). The invasion of XN0422-ZYX and XN0422-Ctrl cells was also decreased by WNK463 (Fig. S5E). These results implied that WNK1/SNAI1 pathway was involved in the regulation of GC invasion. Through the mouse abdominal metastasis experiment, we further noticed a decrease in metastatic foci by WNK463 treatment (Fig. S5F). HE staining confirmed that the metastatic tumor originated from GC cells (Fig. S5G). Interestingly, we also noticed that high and low expression of ZYX could increase and decrease phosphorylation of AKT (S473), respectively (Fig. S5H).

Altogether, our studies revealed that GC patients with high ZYX had a worse prognosis. ZYX might regulate EMT in GC through the WNK1/SNAI1 axis to promote the invasion and metastasis of GC cells, which was reversed by pharmaceutical inhibition of WNK1. Therefore, ZYX/WNK1 could be potential therapeutic targets for the treatment of GC.

## Ethics declaration

All patients in this study signed an informed consent form, and the subject was approved by the Ethics Committee of Southwest Hospital of Army Military Medical University. All animal experiments were approved by the Institutional Animal Care and Use Committee of the Southwest Hospital in accordance with the Guide for the Care and Use of Laboratory Animals.

## Author contributions

Designing research studies: Y.W., Y.H.C., and X.W.C.; Conducting experiments: J.Y., D.F.X., X.M.W., M.M.H., and X.L.G.; Acquiring data: Y.Q., X.X.Y., Z.X.Y., and Y.R.; Analyzing data: Y.W., X.W.B., Y.H.C., and X.W.C.; Writing the manuscript: Y.W., Y.H.C., and X.W.C.

## Conflict of interests

The authors declare that they have no conflict of interests.

## Funding

This work was supported by the Chongqing Academician Program (No. cstc2019yszx-jcyjX0008 to Y.W.) and The Subject of Health Commission of Hubei Province, China (No. WJ2021M222 to X.-M.W.).

## Figures and Tables

**figs1:**
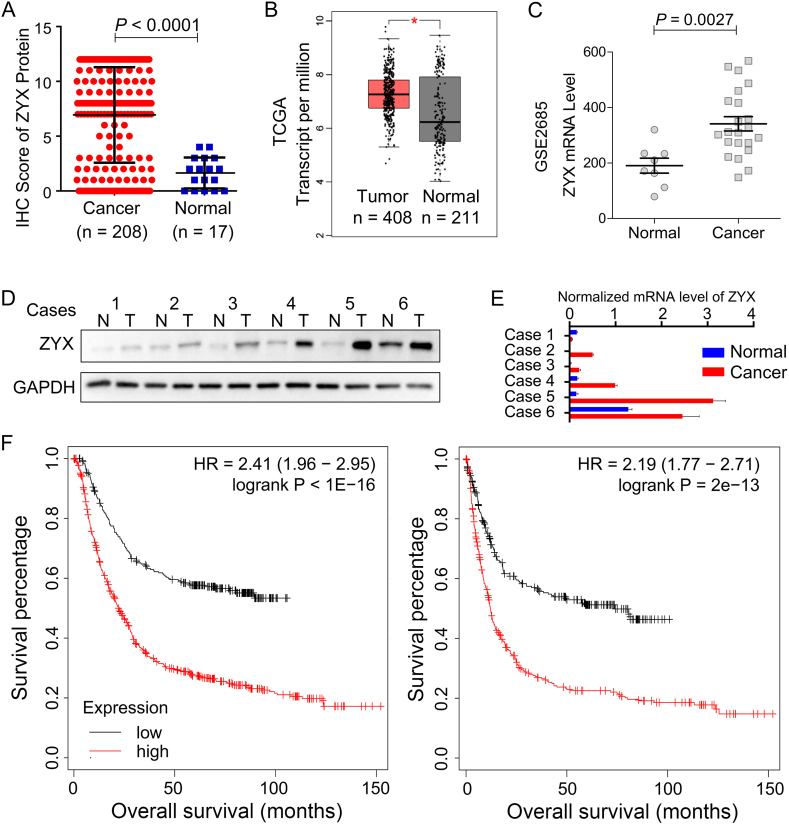


**figs2:**
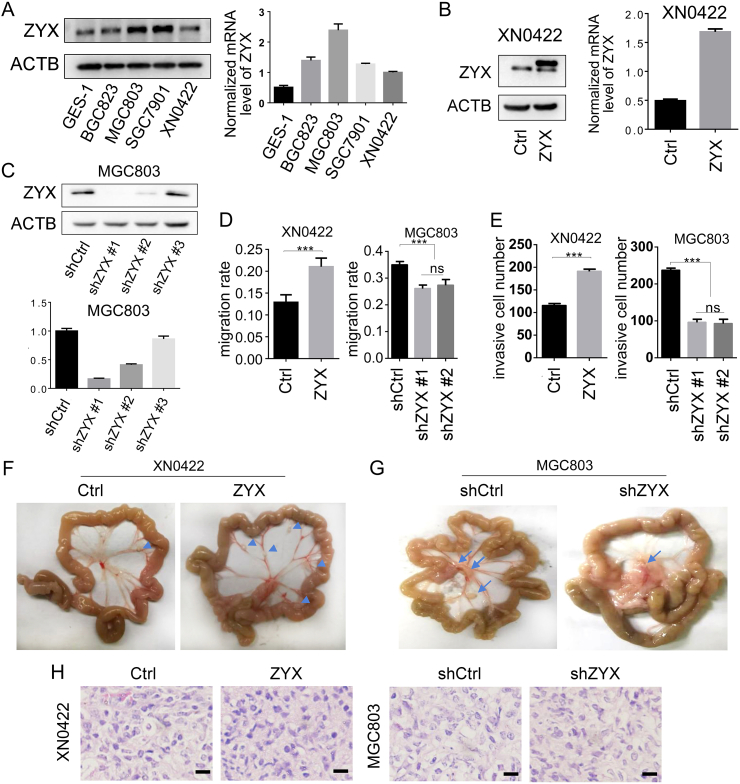


**figs3:**
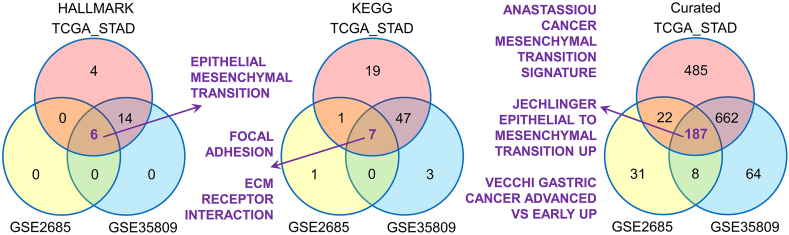


**figs4:**
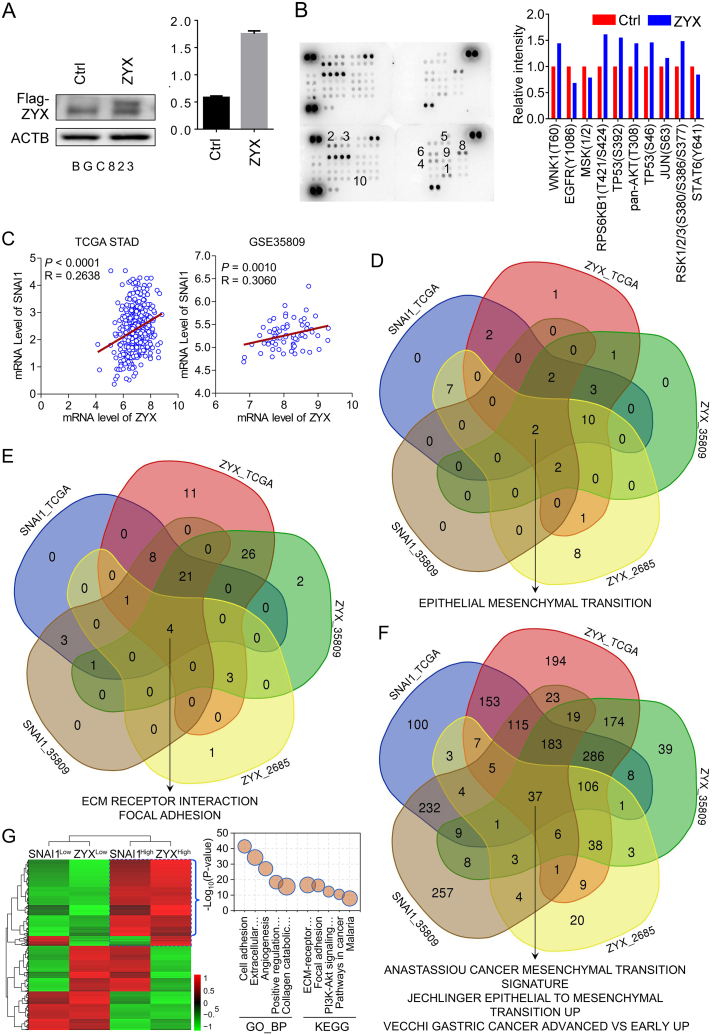


**figs5:**