# Supplementary material for: ZYX promotes invasion and metastasis of gastric cancer cells via WNK1/SNAI1axis
Source: Genes Dis. 2023 Apr 13;11(2):564–7. doi: 10.1016/j.gendis.2023.03.018 (PMC10491912; doi:10.1016/j.gendis.2023.03.018)
Supplement: Multimedia component 1 [file mmc1.docx]

**Supplementary Figure Legends**

**Figure S1. ZYX is highly expressed and predicts poor outcome in GC tissues.**

A) The statistical results of IHC scores in GC and paracancerous tissues. B) and C) mRNA level of zyxin in GC and paracancerous tissues from TCGA_STAD and GSE2685 databases. D) Proteins expression of protein in 6 pairs of resected fresh GC (T) and paracancerous tissues (N). E) mRNA expression of 6 pairs of resected fresh GC and paracancerous tissues. F) Kaplan-Meier survival analysis of KM-Plot data according to mRNA level of ZYX.

**Figure S2. ZYX promotes invasion and metastasis of GC cells.**

A) Protein and mRNA levels of ZYX in gastric cell lines (GES-1, BGC823, MGC803, and SGC7901) and primary gastric cancer cell (XN0422). B) Protein and mRNA levels of ZYX in XN0422-Ctrl and XN0422-ZYX cells. C) Protein and mRNA levels of ZYX in MGC803-shCtrl and MGC803-shZYX cells. D) Scratch tests on migration ability of ZYX *vs.* Ctrl group or shZYX *vs.* shCtrl group. E) Transwell invasion experiments on invasion ability of ZYX *vs.* Ctrl group or shZYX *vs.* shCtrl group. F) Representative images and statistic graph of intraperitoneal metastatic nodules in mice (ZYX *vs.* Ctrl group). G) Representative images and statistic graph of intraperitoneal metastatic nodules in mice (shZYX *vs.* shCtrl group). H) HE staining of indicated cells.

**Figure S3. ZYX expression is correlated with EMT-related genesets.**

Venn diagram of gensets enriched by ZYX high expression from different gastric databases in the context of HALLMARK, KEGG, and Curated, respectively.

**Figure S4. ZYX induced activation of WNK1 and upregulation of SNAI1.**

A) Protein and mRNA levels of ZYX in BGC823-Ctrl and BGC823-ZYX overexpression cells. B) Phosphokinase array image and statistic graph of altered phosphorylated kinases by in BGC823-Ctrl and BGC823-ZYX cells.

C) Pearson correlation of ZYX and SNAI1 in TCGA_STAD and GSE35809 gastric cancer datasets.

D)-F)Venn diagram of gensets enriched by high expression of ZYX and SNAI1 from different gastric databases in the context of HALLMARK (D), KEGG (E), and Curated (F), respectively. G) Left: Heatmap cluster of transcriptomic profiles of low and high expression of ZYX and SNAI1. Right: Top five enriched genesets of co-upregulated genes by ZYX and SNAI1 in the context of Gene Ontology Biological Processes and KEGG Pathways through David analysis.

**Figure S5. Knockdown of WNK1 or pharmaceutical inhibition of WNK1 kinase repressed invasion and metastasis of GC cells.**

A) Knockdown efficiency verification of siRNA targeting WNK1. B) Western blotting of indicated proteins with or without WNK1 siRNA in XN0422-Ctrl and XN0422-ZYX cells. C) Statistic graph of invasive cells of XN0422-Ctrl group and XN0422-ZYX group with or without WNK1 siRNA. D) Western blotting of indicated proteins with or without WNK463 (WNK1 inhibitor) in XN0422-Ctrl and XN0422-ZYX cells. E) Statistic graph of invasive cells of XN0422-Ctrl group and XN0422-ZYX group with or without WNK463. F) Representative images and statistic graph of intraperitoneal metastatic nodules in mice (shCtrl-PBS group, shCtrl-WNK463 group, shZYX-PBS group, and shZYX-WNK463 group). G) Representative HE staining of mouse abdominal metastasis. H) The phosphorylated levels of AKT S473 in XN0422-ZYX *vs.* XN0422-Ctrl group or in MGC803-shZYX *vs.* MGC803-shCtrl.

**Supplementary Materials and Methods**

**Tissue samples of gastric cancer**

GC cohort (n = 208) including 17 non-tumor tissues came from Southwest Hospital of Army Military Medical University. The patients did not receive anti-tumor treatment before operation, and the pathological diagnosis was completed by the Department of Pathology of Army military Medical University. There were 50 female patients and 158male patients with gastric cancer. The TNM staging criteria of the patients were according to the 8th edition of AJCC (American Joint Committee on Cancer) guidelines. Gastric cancer tissue was made into tissue microarray by pathologist. All patients in this study signed an informed consent form, and the subject was approved by the Ethics Committee of Southwest Hospital of Army military Medical University. Fresh gastric cancer and paracancerous tissue were derived from Southwest Hospital of Army military Medical University. The patients did not undergo anti-tumor treatment before operation. The pathological diagnosis was completed by the Department of Pathology of Army military Medical University. All patients signed informed consent.

**Western blotting**

Western blotting was conducted as previously described ^1^. The primary antibodies used in this study were as follows: anti-ZYX (Cat. no. ab109316, Abcam) or anti-GAPDH (Cat. no. 5174, CST) or anti-ACTB (Cat. no. 3700, CST) or anti-WNK1 (Cat. no. 4979, CST) or anti-p-WNK1 (Cat. no. 4946, CST) or anti-CDH2 (Cat. no. 13116, CST) or anti-SNAI1 (Cat. no. 3879, CST) or anti-CDH1 (Cat. no. 14-3249-82, Invitrogen). Phosphate kinase antibody array was ordered from CST and used as manufacturer instruction.

**Cell culture**

Human gastric cancer cell lines BGC823, MGC803, SGC7901 and normal gastric mucosal epithelial cells (GES-1) purchased from the Shanghai Institute of Biochemistry and Cell Biology, XN0422 were isolated from southwest hospital. Cell culture was conducted as previously described.

**Immunohistochemistry and scoring.**

The antibody ZYX used for IHC staining was conducted as previously described, was obtained from Abcam (1:200 dilution) and was scored double-blindly by two professional pathologists. Then, the results of immunochemical staining of gastric cancer tissues were scored by two senior pathologists. The ROC curve was drawn with SPSS20.0, and the area under the ROC curve was 0.730, which was statistically significant (*P* < 0.0001). The cutoff value was calculated to be 6.5 (the highest specificity and sensitivity at this value). According to the cutoff value, the gastric cancer patients with IHC score ≥ 7.0 were defined as high expression of ZYX (ZYX^High^), while the gastric cancer patients with score < 7.0 were defined as low expression of ZYX (ZYX^Low^).The scoring criteria were shown in the table below. Calculation formula: IHC score = staining intensity × proportion of stained cells

| staining intensity |  | proportion of stained cells | | | |
| --- | --- | --- | --- | --- | --- |
|  |  | 1 (5%-24%) | 2 (25%-49%) | 3 (50%-74%) | 4 (75%-100%) |
| 0 (no staining) | score | 0 | 0 | 0 | 0 |
| 1 (weak staining) |  | 1 | 2 | 3 | 4 |
| 2 (medium staining) |  | 2 | 4 | 6 | 8 |
| 3 (strong staining) |  | 3 | 6 | 9 | 12 |

**qRT-PCR**

qRT-PCR was performed as previously described ^2^. The PCR primers were as follows:

ACTB-F: 5′-CATGTACGTTGCTATCCAGGC-3′;

ACTB-R: 5′-CTCCTTAATGTCACGCACGAT-3′;

ZYX-F: 5′-TCTCCCGCGATCTCCGTTT-3′;

ZYX-R: 5′-CCGGAAGGGATTCACTTTGGG-3′.

**Lentivirus infection.**

Lentiviral infection was performed as previously described. The design and packaging of overexpressed lentivirus was completed by Shanghai Hanheng Biotechnology Co., Ltd., and the vector used was pHBLV-CMV-MCS-3flag-EF1-puro. The design and packaging of the lentivirus is undertaken by Shanghai Jikai Co., Ltd. the original sequence of the virus is hU6-MCS-CMV4-Puror, and the viral vector is GV112. The shRNA sequences were listed as below:

shZYX-1: 5′-TCCACATGAAGTGTTACAA-3′;

shZYX-2: 5′-GTTCCAAGTCCAGTACCAA-3′;

shCtrl: 5′-TTCTCCGAACGTGTCACGT-3′.

At 48 hours after virus infection, the medium containing puromycin (5ug/ul) was added to screen.

**Scratch healing experiment.**

The wound healing assay was performed as previously described. After the cell convergence degree reached 90%, the head of the 10ul gun was scratched, washed twice with PBS, and then serum-free RPMI 1640 medium, was added to the 24-hour living cell workstation to take pictures at the same position at 0 h and 24 h, respectively, and the scratch migration rate was calculated by Image J software. Calculation formula: (central area of scratches before cell migration-central area of scratches after cell migration) / central area of scratches before cell migration.

**Matrigel-transwell invasion**

After gastric cancer cells were treated with Transwell invasion assays were performed as previously described. WNK463 (10 mM) for 24 hours, XN0422 cells and MGC803 cells were diluted with serum-free RPMI1640 medium into 1 × 10^6^ Universe 24-well plate with 700 μl containing 10% RPMI1640 medium, and transwell chamber (8-mm pore size, Millipore) upper membrane was added with 10μl RPMI1640 medium and Matrigel mixture solution (Matrigel:RPMI1640 = 1:3), dried and put into a 24-well plate, and then incubated in a cell incubator for 24 hours. Fixed with paraformaldehyde and stained with crystal violet, the upper membrane cells of the transwell chamber were wiped off with a cotton swab, and then naturally air-dried and placed under a 100 × microscope, five visual fields were randomly selected to take pictures.

**Abdominal metastasis experiment in mice**

4-week-old female non-obese diabetic / severe combined immunodeficiency mice were selected and resuspended into 4 × 10^5^ / 200 μl with PBS. The 200ul cell suspension was injected into the abdominal cavity of the mice. After 5 days, the mice were given WNK463 for 5 days (3 cycles). After 6 weeks, the mice were killed to count the number of metastatic tumors in the abdominal cavity. All animal experiments were approved by the Institutional Animal Care and Use Committee of the Southwest Hospital in accordance with the Guide for the Care and Use of Laboratory Animals.

**WNK1-siRNA transfection**

The siRNA was configured as 20 μM. the 2 ml antibiotic-free RPMI1640 medium was added to the 6-well plate with a cell confluence degree of 70%, and the transfection reagents (150 μl OPTI-MEM, 10 μl siRNA, 150 μl OPTI-MEM, 9 μl RNAi MIX),) were put into the cell incubator for 48 hours. We transfected three separate siRNAs targeting WNK1 into XN0422 cells and found that knock-down efficiency of siRNA #1 was the highest, which was used for further experiments.

**Statistical analysis**

The expression of ZYX in gastric cancer and paracancerous tissues was analyzed by online database TCGA (<http://gepia.cancer-pku.cn/detail.php?gene=zyx>). The relationship between the expression of ZYX and the prognosis of patients with gastric cancer was analyzed by KMPLOT database (<http://kmplot.com/analysis/>). All the experiments were repeated 3 times. Spss20.0 was analyzed by t-test, and the cutoff value of immunohistochemical staining was analyzed by spss20.0 analysis. Chi-square test was used to analyze the correlation between the expression of ZYX and pathological parameters of patients with gastric cancer. Univariate and multivariate variances were used to analyze whether the expression of ZYX was an independent prognostic factor in patients with gastric cancer. Kaplan-Meier method was used to analyze the relationship between the expression of ZYX and the overall survival time (OS) and progression-free survival (PDS) in patients with gastric cancer. *P* < 0.05 was considered as statistically significant.

1. Li Y, He ZC, Zhang XN, et al. Stanniocalcin-1 augments stem-like traits of glioblastoma cells through binding and activating NOTCH1. *Cancer Lett.* 2018;416:66-74.

2. Zou H, Chen Q, Zhang A, et al. MPC1 deficiency accelerates lung adenocarcinoma progression through the STAT3 pathway. *Cell Death Dis.* 2019;10(3):148.
