# Supplementary material for: ZYX promotes invasion and metastasis of gastric cancer cells via WNK1/SNAI1axis
Source: Genes Dis. 2023 Apr 13;11(2):564–7. doi: 10.1016/j.gendis.2023.03.018 (PMC10491912; doi:10.1016/j.gendis.2023.03.018)
Supplement: Multimedia component 2 [file mmc2.docx]

**Table S1. Relationships of ZYX protein level with clinicopathological features of gastric cancer patients.**

| Clinicopathological Features | | Case Number | | | *P* Value |
| --- | --- | --- | --- | --- | --- |
|  |  | **Total** | **ZYX**^Low^ **(%)** | **ZYX**^High^ **(%)** |  |
| Age (years) | ≤ 60 | 118 | 40 (33.9) | 78 (66.1) | 0.799 |
|  | > 60 | 90 | 29 (32.2) | 61 (67.8) |  |
| Gender | Female | 50 | 17 (34.0) | 33 (66.0) | 0.887 |
|  | Male | 158 | 52 (33.0) | 106 (67.0) |  |
| Differentiation Grade | High | 25 | 10 (40.0) | 15 (60.0) | 0.729 |
|  | Middle | 70 | 22 (31.4) | 48 (68.6) |  |
|  | Low | 113 | 37 (32.4) | 76 (62.3) |  |
| Tumor Size (cm) | ≤ 3 | 83 | 37 (44.6) | 46 (55.4) | 0.015 |
|  | 3-6 | 91 | 22 (24.2) | 69 (75.8) |  |
|  | > 6 | 34 | 10 (29.4) | 24 (70.6) |  |
| Tumor Recurrence | Yes | 86 | 41 (47.7) | 45 (52.3) | 0.000 |
|  | No | 122 | 28 (23.0) | 94 (77.0) |  |
| T Stage | T1 | 16 | 9 (56.3) | 7 (43.7) | 0.002 |
|  | T2 | 38 | 16 (42.1) | 22 (57.9) |  |
|  | T3 | 65 | 27 (41.5) | 38 (58.5) |  |
|  | T4 | 89 | 17 (19.1) | 72 (80.9) |  |
| N Stage | 0 | 72 | 35 (48.6) | 37 (51.4) | 0.001 |
|  | 1 | 37 | 11 (29.7) | 26 (70.3) |  |
|  | 2 | 48 | 16 (33.3) | 32 (66.7) |  |
|  | 3 | 51 | 7 (13.7) | 44 (86.3) |  |
