# Supplementary material for: ZYX promotes invasion and metastasis of gastric cancer cells via WNK1/SNAI1axis
Source: Genes Dis. 2023 Apr 13;11(2):564–7. doi: 10.1016/j.gendis.2023.03.018 (PMC10491912; doi:10.1016/j.gendis.2023.03.018)
Supplement: Multimedia component 3 [file mmc3.docx]

**Table S2. Relationship of ZYX protein level with prognosis of gastric cancer patients by ANOVA.**

| **Clinicopathological Features** | **One-way ANOVA** | | **Multivariate ANOVA** | |
| --- | --- | --- | --- | --- |
|  | **HR (95% CI)** | ***P* Value** | **HR (95% CI)** | ***P* Value** |
| Age (≤ 60 years) | 0.703 (0.475-1.040) | 0.078 | / | / |
| Gender (Male) | 1.487 (0.944-2.342) | 0.087 | / | / |
| Differentiation | 1.466 (1.091-1.970) | 0.011 | 1.607 (1.177-2.195) | 0.403 |
| T Stage | 2.100 (1.617-2.728) | 0.000 | 1.283 (0.975-1.688) | 0.000 |
| N Stage | 1.712 (1.447-2.025) | 0.000 | 1.465 (0.760-2.822) | 0.000 |
| ZYX^High^ | 2.800 (1.688-4.644) | 0.000 | 2.452 (1.467-4.098) | 0.001 |
